# Clinical outcomes of GLP-1 receptor agonists VS placebo among patients with heart failure with preserved ejection fraction: a systematic review and meta-analysis

**DOI:** 10.1186/s43044-026-00793-w

**Published:** 2026-11-14

**Authors:** Edward Davis, Leonardo Alexander, Nikolaus Tobian, Fernando Alonzo Theja, Ardeno Kristianto, Linawati Hananta

**Affiliations:** 1 Department of Pharmacology, School of Medicine and Health Sciences, Atma Jaya Catholic University of Indonesia, Jakarta, Indonesia; 2 School of Medicine and Health Sciences, Atma Jaya Catholic University of Indonesia, Jakarta, Indonesia; 3 Department of Internal Medicine, St. Carolus Hospital, Jakarta, Indonesia

**Keywords:** Glucagon-like peptide 1 receptor agonist, semaglutide, Heart failure preserved ejection fraction

## Abstract

**Background:**

Heart failure with preserved ejection fraction (HFpEF) is a complex form of heart failure which is marked by impaired diastolic function. It has complex pathophysiology involving systemic inflammation, myocardial fibrosis and arterial stiffness. The existence of other comorbidities namely obesity and diabetes further worsens these processes. Currently there is limited treatment option for HFpEF.

**Methods:**

Systematic search from 5 databases was conducted to identify studies that evaluated the impacts of glucagon-like peptide-1 receptor agonist (GLP-1 RA) on the clinical outcomes of HFpEF. Quantitative analysis was performed using random effect model were performed on clinical outcomes. Results are presented as odds ratio (OR) along with its 95% confidence interval.

**Results:**

3 Studies involving 1463 patients were included. GLP-1 RA reduced NT-pro Brain Natriuretic Peptide (NT-pro BNP) levels and enhanced 6-minute walking test, Kansas City Cardiomyopathy Questionnaire clinical summary score. Significant reduction in adjudicated heart failure event was observed (OR 0.32, 95% CI: 0.15–0.67, *p* < 0.05, I^2^ = 5%). No significant difference in all-cause mortality (OR 0.63, 95% CI: 0.27–1.48, *p* = 0.29, I2 = 0%) and cardiovascular mortality (OR 0.27, 95% CI: 0.04–1.66, *p* = 0.16, I2 = 0%) were observed.

**Conclusion:**

GLP-1 RA was effective in improving quality of life parameters and lowered the risk of cardiovascular events of HFpEF patients; however it did not significantly reduce mortality in those who suffered from HFpEF.

## Introduction

 Heart failure with preserved ejection fraction (HFpEF) is a complicated medical condition marked by the presence of diastolic dysfunction, in which the heart has difficulty relaxing and filling with blood effectively. This would lead to significantly higher filling pressures in the ventricle and ultimately heart failure symptoms [1]. HFpEF is also driven by various other abnormalities, such as systemic inflammation, endothelial dysfunction, myocardial fibrosis, and increased arterial stiffness. Other comorbidities such as type 2 diabetes mellitus and obesity will further exacerbate these pathological processes, further complicating disease management [2]. The global prevalence of HF is currently estimated to be around 1–3% of the total population [3]. In the US, the lifetime risk of developing HF for individuals who aged over 45 years old ranges from 20% to 45% [4]. Based on the data from ESC Long-Term Registry database in 2017, 60% of HF patients suffered heart failure with reduced ejection fraction (HFrEF, EF < 40%), 24% heart failure with mild reduced ejection fraction (HFmrEF, EF 40–49%), and 16% as heart failure with preserved ejection fraction (HFpEF, EF$\:\ge\:$50%) [5].

Current therapeutic strategies for HFpEF remain limited, focusing mainly on symptom relief and management of comorbidities. Unlike HFrEF, HFpEF lacks therapies that consistently reduce morbidity and mortality [6]. HFpEF is challenging to treat due to its multifactorial pathophysiology, highlighting the need for novel therapies. Recent evidence suggests that glucagon-like peptide-1 receptor agonists (GLP-1 RAs), which was initially developed for type 2 diabetes, may benefit HFpEF through anti-inflammatory effects, improved endothelial function, and reduced arterial stiffness and fibrosis. GLP-1 RAs have also shown potential to improve exercise capacity, particularly in patients with obesity and metabolic disorders [7].

GLP-1 RA works by increasing insulin production and decreasing inappropriate secretion of glucagon, it can also prolong satiety through its ability to prolong gastric emptying [8]. It was first approved by the Food and Drug Administration for type 2 diabetes in 2005 [9]. Current recommendations suggests that it is also useful as obesity treatment [10]. GLP-1RAs have long been used to improve glycemic control and promote weight loss in patients with type 2 diabetes, particularly those with overweight or obesity [11]. Furthermore, GLP-1RAs may provide cardioprotective effects by reducing major adverse cardiovascular events (MACE) in patients who suffered from atherosclerotic cardiovascular disease [12], and potentially preventing hospitalization caused by heart failure decompensation [13].

Previous clinical trial reported that GLP-1RA had improved diastolic function. GLP-1RA reduced early left ventricle (LV) diastolic filling and LV filling pressure, thus offloading the LV [14]. Furthermore, GLP-1RAs also promote weight loss, which is particularly important in HFpEF, as approximately 80% of these patients are obese. Obesity is one of the key factors that contributed to the establishment and progression of HFpEF [15]. A study showed that even in non-HF patients who suffered morbid obesity, cardiac remodeling, lower left ventricle end-diastolic volume, lower cardiac output were observed [16]. Current data suggests that GLP-1RAs can be benefitting clinical outcomes in HFpEF.

Previous meta-analysis evaluated the effects of GLP-1RAs in patients with HFrEF and demonstrated potential improvements in clinical and hemodynamic parameters [17]. However, current latest evidence on the use of GLP-1RAs in HFpEF patient is still limited [18]. Given the distinct pathophysiology between HFrEF and HFpEF, further research are required to evaluate whether GLP-1RAs provide similar clinical benefits or exert unique mechanisms in the HFpEF population.

## Methods

This systematic review and meta analysis was performed by adhering to the Preferred Reporting Items for Systematic Reviews and Meta-Analyses (PRISMA). The protocol of this meta-analysis has been previously registered in the international prospective register of systematic review (PROSPERO), registration ID: CRD42024618265. Literature search was performed systematically on Sage, ProQuest, ScienceDirect, Pubmed, Ebsco in November 2024 without publication date restriction. The following search terms were used: (glucagon-like peptide 1 receptor agonist or GLP-1 agonist or GLP-1 analogs) and (heart failure preserved ejection fraction or HFpEF or diastolic heart failure).

**Fig. 1:**
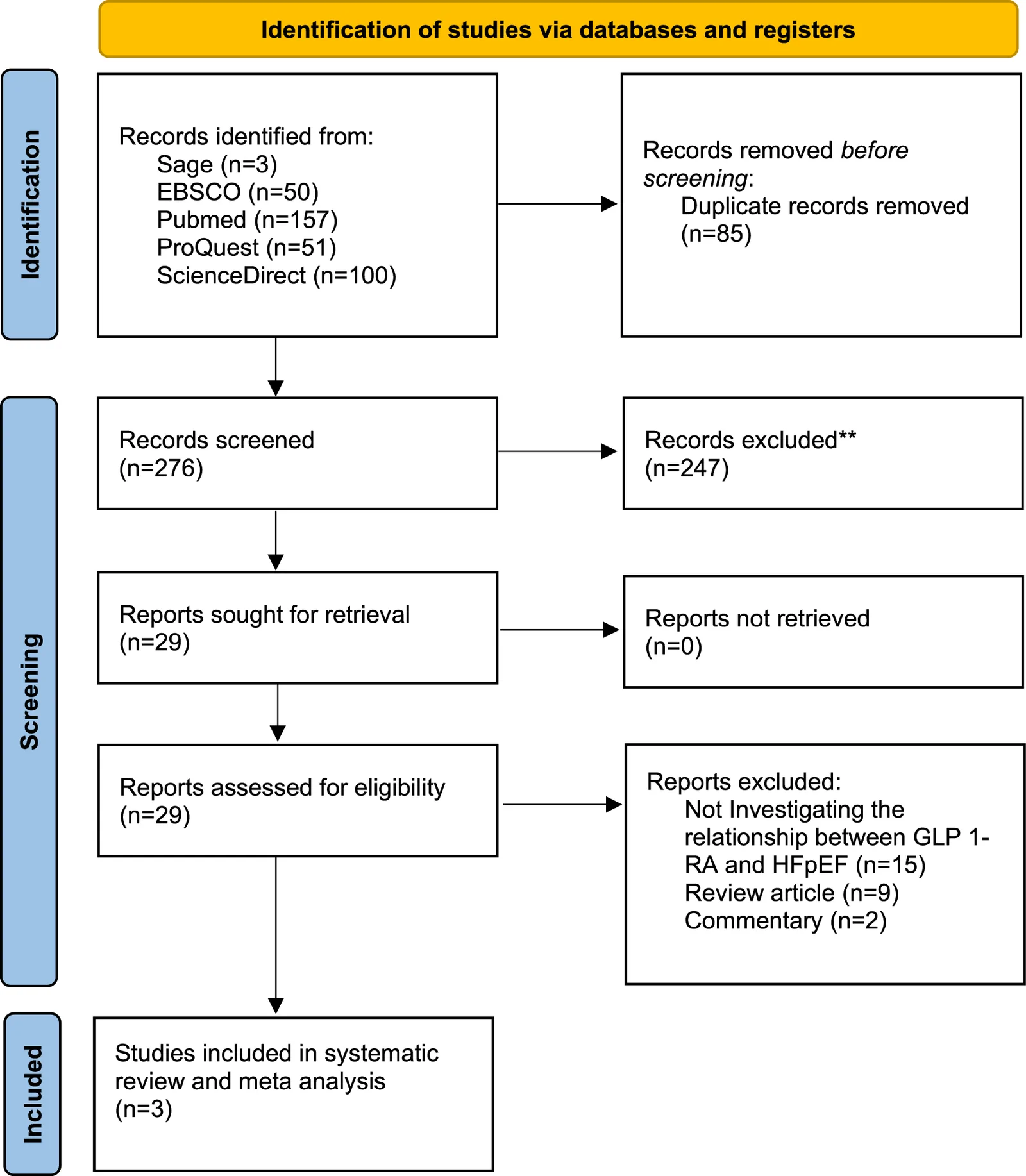
PRISMA flow chart

All identified articles were screened by 2 reviewers who worked independently of each other, any disagreements that arose will be resolved with a third reviewer. Full text review was performed for each eligible article. Articles that passed inclusion criteria were included in this meta-analysis. The flow chart of this meta-analysis can be seen in Fig. 1.

### Inclusion and exclusion criteria

The inclusion criteria for this meta analysis are: clinical trial, cohort studies, case control; comparing the effects of GLP-1 RA vs. placebo in patients with HFpEF. Studies were excluded if the article was not in English; review article; case report; no full text available.

### Risk of bias analysis (ROB analysis)

The assessment of ROB on clinical trials was done using Cochrane risk-of-bias tool for randomized trials (RoB), Cohort study was assessed using Newcastle - Ottawa (NOS) Quality Assessment Scale Cohort Studies. All articles that were included in this meta-analysis were scored by 2 independent reviewers. Differences will be resolved through consensus.

### Quantitative analysis

Forest plot was generated with Review Manager 5.4. Random effects model was utilized in this meta-analysis, since there was substantial study design heterogeneity among the different studies. Odds ratio (OR) was chosen as the measure of effect. Data were presented as OR along with the 95% confidence interval. Heterogeneity was assessed with *I*^2^ statistic. *I*^2^ exceeding 50% suggests that there is substantial heterogeneity. Conversely *I*^2^ below 50% suggests there is no significant heterogeneity. Results with p value of < 0.05 were considered as statistically significant.

## Results

Systematic search with the search terms above yielded a total of 361 articles, following duplicate removal there were 276 articles that underwent title and abstract screening. Twenty nine that passed initial screening were obtained for full text analysis. After full text analysis, three eligible studies were included in our data synthesis. Further information about study characteristics including intervention and outcome in selected studies is provided in Table 1.

**Table 1:** Study characteristics of all article included in this research

Study	Sample size (*n*)	Follow up duration	Age (median/mean)	Population	intervention	Selected outcomes
Rehman et al. [19] (2024)	318	52 weeks	69	Patients with obesity	Semaglutide escalated gradually over 16 weeks and reached maintenance dose of 2.4 mg	6-min walking distance, NT-proBNP level reduction, adjudicated heart failure events
Kosiborod et al. [20] (2023)	529	52 weeks	69	Patients with obesity	Semaglutide, once -weekly dose of 2.4 mg	KCCQ-CSS score improvement, adjudicated heart failure events, 6-min walking distance, cardiovascular mortality, NT-proBNP level reduction,, all cause mortality
Kosiborod et al. [21] (2024)	616	52 weeks	69	Patients with obesity and type 2 diabetes	Semaglutide, once -weekly dose of 2.4 mg	KCCQ-CSS score improvement, cardiovascular mortality, 6-min walking distance, adjudicated heart failure events, NT-proBNP level reduction, all cause mortality

### NT-proBNP reduction

The outcome of GLP-1RA administration on NT-proBNP levels in patients who suffered both obesity and HFpEF remains inconclusive. In a retrospective study, GLP-1 RA treatment demonstrated a trend toward NT-proBNP reduction; however, failed to achieve statistical significance (estimated ratio: 0.80, 95% confidence interval [CI]: 0.56–1.14, *p* = 0.240) [19]. Meanwhile, two other studies reported different results. The STEP-HFpEF trial, which evaluated GLP-1 RA administration in individuals who suffered from concomitant obesity and HFpEF showed a 20.9% reduction in NT-proBNP levels compared to minimal change of only 5.3% reduction in the control group, with an estimated difference ratio of 0.84 (95% CI: 0.71–0.98) [20]. Meanwhile, the administration of GLP-1 RA in people who suffered obesity, diabetes mellitus, and HFpEF (STEP-HFpEF DM) trial revealed that the GLP-1RA group had 23.2% lower NT-proBNP levels versus modest 4.6% decrease observed in the control group (difference ratio: 0.8, 95% CI: 0.7–0.9) [21].

### 6 min walk test

GLP-1 RA therapy consistently led to notable enhancements in functional capacity, as reflected by six-minute walk test (6MWT), across multiple studies. A retrospective cohort study demonstrated a mean increase of 15.1 m (95% CI: 5.8–24.4, *p* = 0.002) in the GLP-1 RA-treated participants in comparison to placebo group. This finding was further supported by two large-scale RCTs, wherein GLP-1 RA administration resulted in a mean increase of 20.3 m (95% CI: 8.6–32.1, *p* < 0.001) [20] and 14.3 m (95% CI: 3.7–24.9, *p* = 0.008) [21], respectively.

### Kansas city cardiomyopathy questionnaire clinical summary score

The impact of GLP-1 RA on patient-reported symptoms and quality of life, as determined by the Kansas City Cardiomyopathy Questionnaire clinical summary score (KCCQ-CSS), were consistently favorable across multiple studies. A randomized controlled trial (RCT) involving patients with HFpEF, obesity, type 2 diabetes showed a significant increase in KCCQ-CSS scores, on patients who received GLP-1 RA as compared to those who received placebo (MD [mean difference]: 7.8 points, 95% CI: 4.8–10.9, *p* < 0.001) [20]. Similarly, another RCT evaluating GLP-1 RA in a population with concomitant type 2 diabetes and HFpEF observed a comparable improvement in KCCQ-CSS (mean difference: 7.3 points, 95% CI: 4.1–10.4, *p* < 0.001) [21]. These findings indicated that GLP-1 RA significantly alleviated the symptoms and improved quality of life among individuals with obesity-associated HFpEF. This evidence further supports its potential therapeutic benefit beyond glycemic and weight control.

### Adjudicated heart failure event

**Fig. 2:**
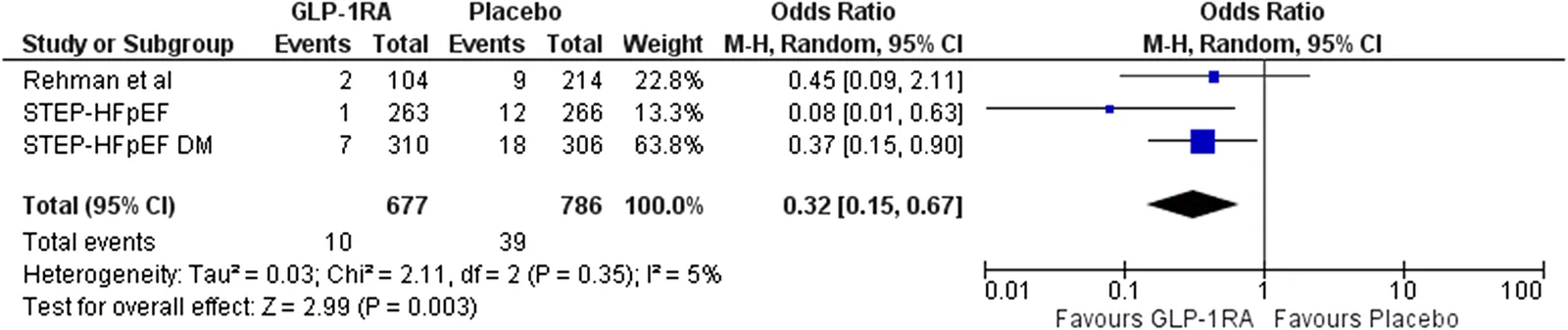
Forrest plot of adjudicated heart failure event

Adjudicated heart failure events (Hospitalization and urgent visit to emergency department) were reported by all 3 studies (Fig. 2). Pool analysis showed that in comparison to group that received placebo, GLP-1RA use significantly lowered the risk of adjudicated heart failure event (hospitalization or urgent visit for heart failure) in HFpEF (pooled OR 0.32, 95% CI: 0.15–0.67, *p* < 0.05, I^2^ = 5%). The I^2^ was 5%, suggesting minimum heterogeneity.

### All-cause mortality

**Fig. 3:**
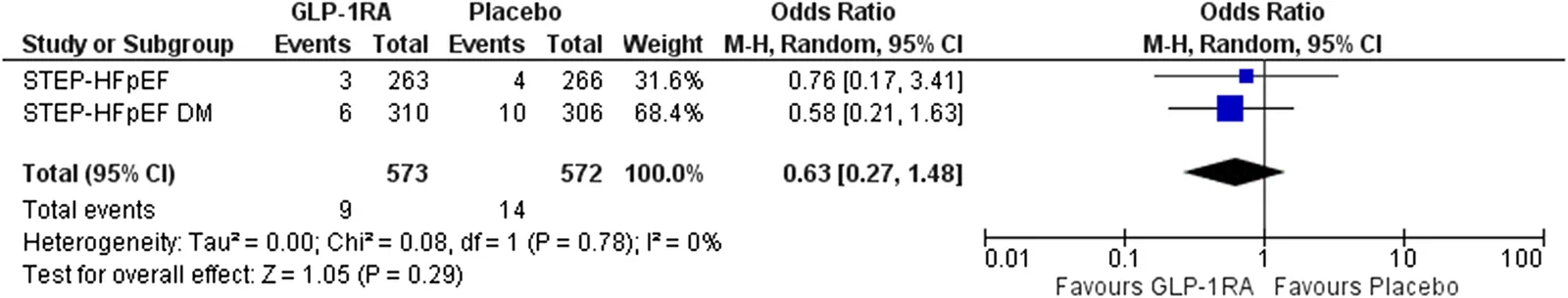
Forrest plot of all cause mortality

Two studies reported the all-cause mortality outcomes, with a pooled odds ratio (OR) of 0.63 (95% CI: 0.27–1.48, *p* = 0.29, I^2^ = 0%), this revealed that there is no statistical significance difference between GLP-1RA group and placebo group in reducing all-cause mortality risk (Fig. 3).

### Cardiovascular mortality

**Fig. 4:**
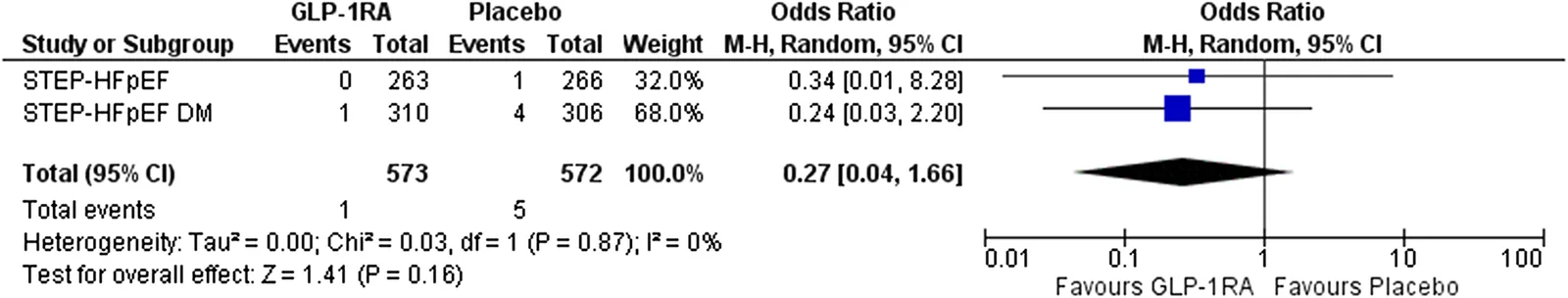
Forrest plot of cardiovascular mortality

We observed no statistically significant reduction in cardiovascular mortality with GLP-1RA use compared to placebo (Fig. 4), with a pooled OR 0.27 (95% CI: 0.04–1.66, *p* = 0.16, I^2^ = 0%).

### Risk of bias assessment

Table 2 displays the bias risk evaluated using the Newcastle-Ottawa scale (NOS) for a cohort study. This risk of bias assessed 3 domains such as selection, comparability, and outcome. Each domain has criteria to get a star that determines the quality of study.

### ROB 1 cochrane-clinical trial

The Cochrane Risk of Bias (RoB 1) tool was developed to examine the internal validity of RCTs. It assesses seven key areas where bias may be introduced such as random sequence generation, concealment of allocation, the presence of selective reporting, other bias, blinding of investigator and participant, blinding assessment outcome, incomplete outcome data. Each of the study will be classified into either low, unclear, or high risk of bias. Table 3 displays the bias risk of studies evaluated using the RoB 1 for RCTs.

**Table 2:** NOS critical appraisal for cohort study

	Selection (one star in each criteria)				Comparability (max 2 criteria)	Outcome( one star in each criteria)			
Study	Representativeness of the exposed cohort	Selection of the non-exposed cohort	Ascertainment of exposure	Outcome absent at start of study	Comparability of cohorts by design or analysis	Assessment of outcome	Follow-up long enough for outcomes	Adequacy of follow-up	Overall grade
Rehman et al.	*	*	*	*	*	*	-	*	good quality

Selection

1. Representativeness of the exposed cohort
2. Selection of the non-exposed cohort
3. Ascertainment of exposure
4. Demonstration that outcome of interest was not present at start of study

Comparability

1. Comparability of cohorts on the basis of the design or analysis controlled for confounders

Outcome

1. Assessment of outcome
2. Was follow-up long enough for outcomes to occur
3. Adequacy of follow-up of cohorts

Good quality: 3 or 4 stars in selection domain AND 1 or 2 stars in comparability domain AND 2 or 3 stars in outcome/exposure domain

Fair quality: 2 stars in selection domain AND 1 or 2 stars in comparability domain AND 2 or 3 stars in outcome/exposure domain

Poor quality: 0 or 1 star in selection domain OR 0 stars in comparability domain OR 0 or 1 stars in outcome/exposure domain

**Table 3:**
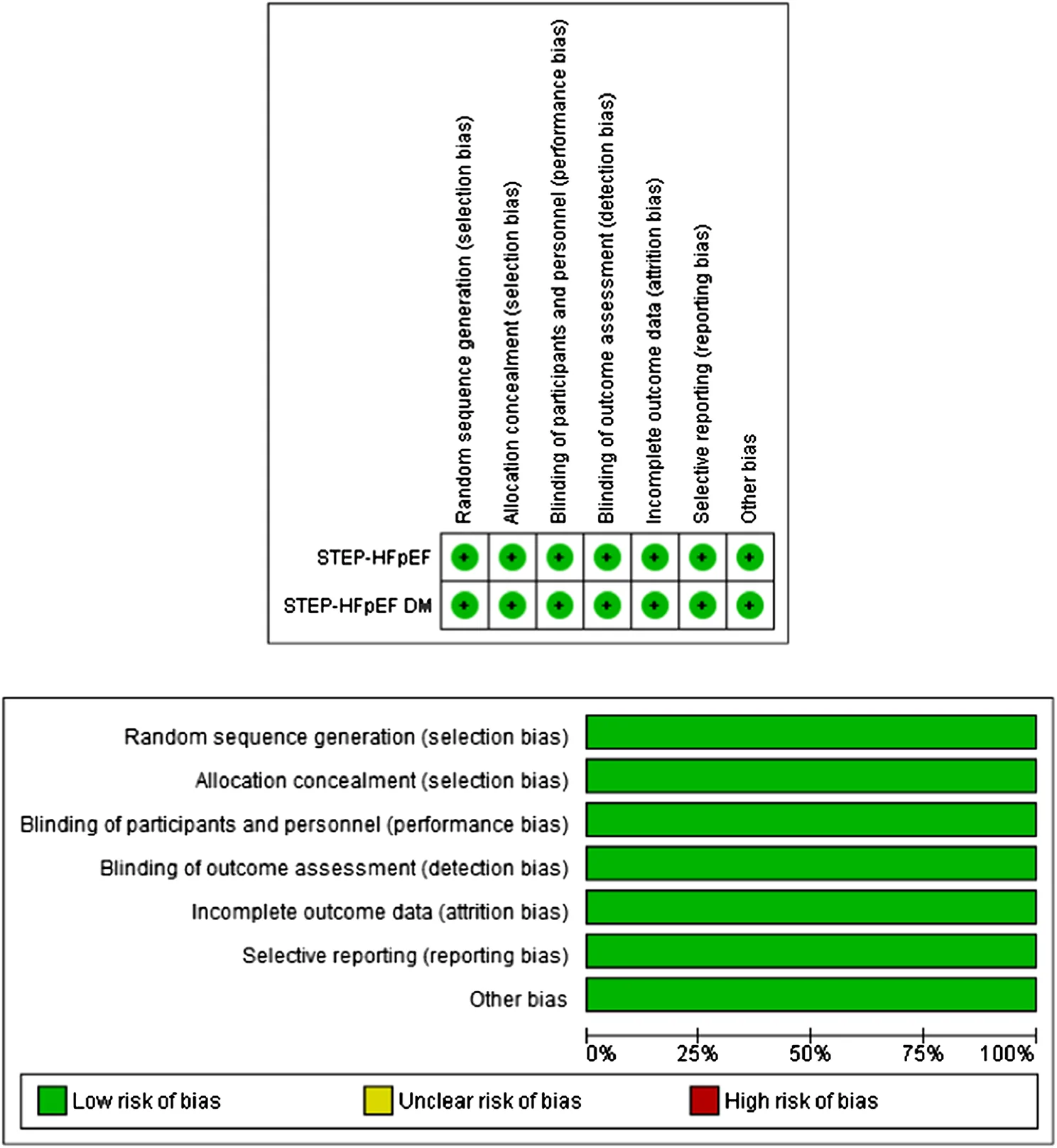
RoB 1 critical appraisal for RCTs studies

## Discussion

Our meta-analysis found that GLP-1RA significantly reduced adjudicated heart failure events in HFpEF; however it was not effective in lowering the risk of cardiovascular or overall mortality. Furthermore, based on qualitative analysis GLP-1RA was also effective in increasing functional capacity and increasing quality of life. All 3 studies included in this study consistently showed that GLP-1RA significantly increased 6MWT, while 2 RCT that measured KCCQ-CSS consistently showed that GLP-1RA improved KCCQ-CSS. The findings for NT-proBNP reduction were inconclusive, two RCT reported reduction of NT-proBNP in patients with GLP-1RA; however 1 study reported that GLP-1RA did not show significant decrease in NT-proBNP levels.

Our meta-analysis found no statistically significant link between GLP-1RA use and reduced cardiovascular mortality in patients with HFpEF. One possible explanation is that the relatively low number of cardiovascular death events during the follow-up period limited the ability of the included trials to detect differences in mortality [22, 23]. Differences in patient characteristics and underlying risk profiles may also contribute to variation in mortality outcomes among patients with HFpEF [24]. Cardiovascular mortality in HFpEF has been shown to rise progressively, with reported rates of 15%, 31%, 47%, and 74% at 1, 3, 5, and 10 years, respectively [25], respectively, while 1- and 5-year survival rates are 61% and 59% [26]. Key mortality predictors include tricuspid regurgitation, presence of diabetes, malignancy, and being male [25]. The studies in our analysis only followed patients for 52 weeks, a duration that may be insufficient to observe mortality outcomes. Longer-term trials are necessary to clarify GLP1-RA effects in this population. Given the increasing prevalence of HFpEF relative to HFrEF [27], further research is essential to define effective therapies.

Our study also found that GLP-1 RA therapy in patients with HFpEF was associated with improvements in functional capacity, as reflected by the 6WMT, and in quality of life, as assessed by the KCCQ-CSS. Although these findings indicate potential clinical advantages of GLP-1 RA in this population, the extent to which these improvements reflect direct cardiac effects remains to be clarified. Considering the significant weight reduction associated with GLP-1 RA treatment [28, 29] and the known association between obesity and reduced functional capacity in HFpEF [30, 31], it is also possible that the observed benefits can be partially attributed to reductions in body weight. In individuals with HFpEF, weight loss has been known to be associated with enhanced exercise tolerance [32], reduced ventricular filling pressures [33], and overall symptom relief. Therefore, weight loss represents one plausible contributor to the observed improvements in 6MWT and KCCQ scores, which leads to reduced cardiac workload and improved peripheral oxygen delivery, rather than by myocardial effects of GLP-1RA [32, 33]. It remains unclear to what extent these benefits are attributable to weight loss, direct cardiovascular effects of GLP-1RA, or a combination of both. Further research should seek to distinguish the weight-related effects of GLP-1 RAs from those that occur independently of weight loss.

Large body of evidence have demonstrated the beneficial effects of GLP-1RA on HF. In patients who suffered from type 2 diabetes mellitus, particularly those who suffered or have elevated risk for coronary artery atherosclerosis, and HF, GLP-1RAs such as albiglutide, semaglutide, dulaglutide, and liraglutide have been linked to decreased incidence of MACE [34]. Regarding its pharmacodynamic effects, several studies have shown that GLP-1 RA has a dose-dependent relationship. The dose is directly proportional to the effectiveness in decreasing HbA1c levels and body mass. For instance, in the STEP clinical trial, 2.4 mg of semaglutide per week reduced mean weight by 14.9% to 17.4% among individuals with excess body weight or obesity without type 2 diabetes, over 68 weeks of treatment. In addition, more than 84% of participants achieved full semaglutide dosing by week 68, indicating good tolerability of dose escalation. In another study, semaglutide 2.4 mg also caused a weight loss of 15.2% compared to only 2.6% on placebo after 104 weeks of treatment [35, 36]. These findings provide evidence for the weight-reducing effects of semaglutide, however, they do not establish that weight loss directly mediates the improvements in functional capacity or quality of life observed in patients with HFpEF.

GLP-1 RA are generally well tolerated and have a high safety profile. Common side effects associated with semaglutide were adverse gastrointestinal effects, such diarrhea and nausea, which were mostly mild to moderate and could be minimized with gradual dose titration. Tapering off is generally not necessary since GLP-1 RAs are not classified as agents that cause withdrawal symptoms. Temporary dose reduction to the initial dose before titration may be considered should side effects occur. It is important to note that GLP-1 RA may cause an increase in heart rate, which can be harmful in HF and their safety profile in patients who have HFrEF remains not well established [34]. Close monitoring is advised in those patients, in order to prevent potential adverse effects that may arise.

Research on GLP-1 RA utilization was also conducted in patients with various spectrums of left ventricular ejection fraction (LVEF), including HFmrEF and HFrEF. In Butler et al. study, semaglutide was prescribed to a group of respondents divided into different LVEF classification including HFmrEF. Semaglutide was associated with improvements in HF symptoms and functional limitations, as assessed by KCCQ-CSS, as well as improvements in exercise performance(via 6MWD), reductions in body weight, and reductions in inflammatory markers across different LVEF categories [37]. These findings suggest that the clinical effects of semaglutide may extend beyond patients with strictly preserved LVEF; however, the extent to which these effects differ according to LVEF remains to be established.

While there is increasing evidence that suggested that GLP-1 agonists may improve cardiac metabolism and exert cardioprotective effects, a study by Marguiles et al. who used GLP-1RA in patients with HFrEF found contradictory result. This study reported that GLP-1 RA did not provide significant improvement as compared to placebo in improving clinical stability, reducing mortality, rehospitalization rates due to heart failure. It also did not improve NT-proBNP levels, echocardiographic parameters, 6MWT, or KCCQ-CSS. Furthermore, there are potential adverse effects that need to be monitored closely, namely heart rate increase that can worsen symptoms of heart failure. These findings highlight potential differences in treatment effects across the spectrum of LVEF and suggest that results observed in HFpEF should not be directly extrapolated to patients with HFrEF. Further studies are required to clarify the safety and efficacy of GLP-1RA therapy in patients with reduced ejection fraction [38].

The findings of this meta-analysis should be interpreted in the context of several limitations. First, the quantitative synthesis is based on a restricted pool of literature comprising only three eligible studies with different study designs (two randomized controlled trials and one retrospective cohort). The total sample size was only 1,463 patients. This limited sample size heavily constrains the statistical power of the analysis, which is the most probable explanation for the absence of a statistically significant treatment effect on mortality. Both cardiovascular and all-cause deaths were infrequent across the pooled cohort, resulting in wide confidence intervals (cardiovascular mortality OR 0.27, 95% CI 0.04–1.66; all-cause mortality OR 0.63, 95% CI 0.27–1.48) that may reflect an underpowered analysis rather than a definitive lack of survival benefit. Second, the follow-up duration across all included studies was limited to 52 weeks. This restricted timeframe may be insufficient to adequately capture long-term mortality outcomes in the HFpEF population, where survival trajectories typically diverge over multiple years. Finally, because GLP-1 receptor agonists induce significant weight loss, the exact mechanisms behind the observed clinical improvements remain unclear. It is difficult to determine if the enhanced functional capacity and quality of life result from direct cardioprotective effects or are secondary to weight reduction.

## Conclusion

In conclusion, the present study revealed that GLP-1RA was able to reduce adjudicated heart failure events, enhance the quality of life and functional capacities in patients who suffered from HFpEF. Our meta-analysis also showed that GLP-1RAs were not significantly associated with reductions in either cardiovascular mortality or all-cause mortality compared with placebo. However, several limitations should be considered, including differences in study designs, minimal eligible studies, and relatively small sample sizes. Furthermore, the follow-up duration may not have been sufficient to capture long-term mortality outcomes.

## Data Availability

No datasets were generated or analysed during the current study.

## Abbreviations

HFpEF: Heart failure with preserved ejection fraction
GLP-1 RA: Glucagon-like peptide-1 receptor agonist
NT-Pro BNP: NT-pro brain natriuretic peptide
OR: Odds ratio
HFrEF: Heart failure with reduced ejection fraction
HFmrEF: Heart failure with mild reduced ejection fraction
HF: Heart failure
LV: Left ventricle
PRISMA: Preferred reporting items for systematic reviews and meta-analyses
PROSPERO: Prospective register of systematic review
RoB: Risk of bias
NOS: Newcastle ottawa scale
STEP-HFpEF: Semaglutide treatment effects in people with obesity and HFpEF
STEP-HFpEF DM: Semaglutide treatment effects in people with obesity, HFpEF, and diabetes mellitus
6MWT: 6 min walking test
KCCQ-CSS: Kansas city cardiomyopathy questionnaire clinical summary score
RCT: Randomized controlled trial
T2DM: Type 2 diabetes mellitus
ASCVD: Atherosclerotic cardiovascular disease
MACE: Major adverse cardiovascular events
LVEF: Left ventricular ejection fraction
